# Cost-Effective and Highly Photoresponsive Nanophosphor-P3HT Photoconductive Nanocomposite for Near-Infrared Detection

**DOI:** 10.1038/srep16761

**Published:** 2015-11-16

**Authors:** Yi Tong, Xinyu Zhao, Mei Chee Tan, Rong Zhao

**Affiliations:** 1 Engineering Product Development, Singapore University of Technology and Design, 8 Somapah Road, Singapore 487372

## Abstract

The advent of flexible optoelectronic devices has accelerated the development of semiconducting polymeric materials. We seek to replace conventional expensive semiconducting photodetector materials with our cost-effective composite system. We demonstrate in this work the successful fabrication of a photoconductive composite film of poly(3-hexylthiophene-2,5-diyl) (P3HT) mixed with NaYF_4_:Yb,Er nanophosphors that exhibited a ultrahigh photoresponse to infrared radiation. The high photocurrent measured was enabled by the unique upconversion properties of NaYF_4_:Yb,Er nanophosphors, where low photon energy infrared excitations are converted to high photon energy visible emissions that are later absorbed by P3HT. Here we report, a significant 1.10 × 10^5^ times increment of photocurrent from our photoconductive composite film upon infrared light exposure, which indicates high optical-to-electrical conversion efficiency. Our reported work lays the groundwork for the future development of printable, portable flexible and functional photonic composites for light sensing and harvesting, photonic memory devices, and phototransistors.

Continuous efforts have been invested to enhance the conversion of light to electrical signals motivated by the diverse range of emerging technological applications, such as photodetectors, optical communications, sensors, photonic memory, photocatalysts, solar cells, spectroscopy, and phototransistors^1^,^2^,^3^,^4^,^5^,^6^,^7^,^8^. Within the full electromagnetic spectrum, near-infrared (NIR) light has recently garnered rising attention due to the emerging applications in night-vision imaging, biomedical imaging, security, and solar energy conversion^9^,^10^,^11^,^12^,^13^,^14^,^15^,^16^,^17^. To convert photons to electrical signals, most of the traditional materials used are semiconductor materials with direct- or indirect- bandgap, such as silicon, germanium, and III–V materials^18^,^19^,^20^,^21^,^22^,^23^,^24^. Since these semiconductors only absorb photons with energy higher than the semiconductor bandgap, they typically exhibit a weak absorbance in the near-infrared regime. Even though III–V semiconductors can be fine-tuned to absorb more near-infrared light, the cost of III–V materials is high due to the complex and costly physical deposition and epitaxial growth methods. Therefore, a material system that can be made using cost-effective and versatile processing technologies with a high efficiency of conversion of near-infrared light to electrical signals needs to be developed urgently. In addition, the next generation of electronics demands the development of flexible devices (e.g. devices of organic materials) to supersede the conventional semiconductor devices without losing any functions^25^,^26^,^27^,^28^,^29^,^30^,^31^,^32^,^33^,^34^. Although organic semiconductors are used to improve the flexibility of devices, the relatively large bandgap of these organic semiconductors limits the absorption of near-infrared light^35^,^36^,^37^,^38^,^39^,^40^,^41^. Despite the efforts that have been invested towards improving the response of organic semiconductor, such as poly(3-hexylthiophene-2,5-diyl) (P3HT) to NIR light, high photoresponse with P3HT was not achieved^36^,^42^,^43^,^44^,^45^,^46^. To improve the response of organic semiconductor materials to NIR light, one strategy is to use rare-earth (RE) ions doped up-conversion (UC) nanophosphors combined with the specific organic semiconductor film (e.g. P3HT film) with a strong absorption rate of visible lights^47^,^48^. NaYF_4_ is considered as one of the most efficient host for NIR-to-visible conversion due to its low phonon energy and multiple dopants. The key factor is the unique nonlinear UC optical process where high-energy photons are generated by absorbing two or more low-energy near-infrared photons. The resultant high-energy visible emissions are subsequently efficiently absorbed by the organic semiconductor film^49^. Although, a composite P3HT semiconductor polymer film with NaYF_4_:Yb,Er UC nanoparticles was recently reported to have a response to NIR light^47^, the reported photocurrent enhancement was insignificant and hardly useful for device design and fabrication.

Here we report the successful fabrication of the composite P3HT film mixed with NaYF_4_:Yb,Er UC nanophosphors that exhibits a high conversion efficiency of near-infrared light to electrical signals. We have further demonstrated the integration and application of these P3HT-nanophosphor composite films as photoconductive devices. An incredible photocurrent enhancement of ~5 orders is measured under the excitation of near-infrared light with various wavelengths, leading to significant advancements for future design and fabrication of optoelectronics devices. For the next generation of wearable and portable optoelectronics devices, these cost-effective and highly photoresponsive P3HT-nanophosphor composite films with excellent mechanical flexibility promises to be an outstanding candidate.

## Results

### Preparation and characterization of NaYF_4_:Yb,Er core-shell nanophosphors

**Figure 1:**
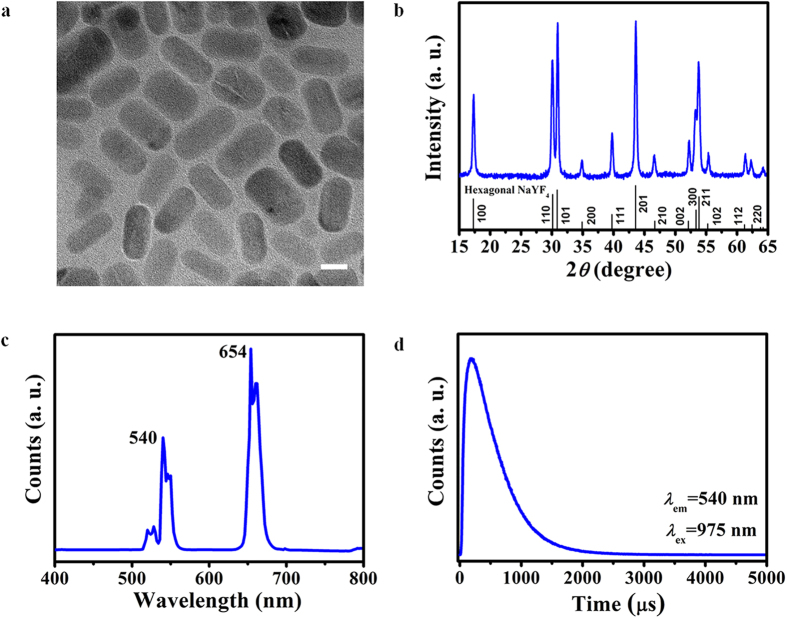
Characterization of our as-synthesized NaYF_4_:Yb,Er core-shell nanoparticles. (**a**) TEM micrograph. Scale bar, 20 nm. (**b**) XRD profile. (**c**) Steady state emission spectrum. (**d**) Time-resolved luminescence spectrum.

Hexagonal phase NaYF_4_:Yb,Er core-shell upconversion nanophosphors (UCN) with excellent visible upconversion emissions upon excitation at 975 nm was synthesized using a thermal decomposition method (see Fig. 1). As shown in Fig. 1a, the TEM micrograph shows that NaYF_4_:Yb,Er UCN is composed of mostly nanorods with a longitudinal size of 34.8 ± 11.4 nm with an aspect ratio of 1.89. The size distribution was measured from the SEM image of NaYF_4_:Yb,Er UCN (Supplementary Fig. 1a) and shown in Supplementary Fig. 1b. The as-synthesized UCN consists of pure hexagonal NaYF_4_ phase (JCPDS 16-0334) (Fig. 1b), where the broad XRD peaks indicate that small crystallites were synthesized. Using the Scherrer equation, the estimated grain size for the UCN was 25.6 ± 1.8 nm. The EDX spectrum (Supplementary Fig. 2 and Supplementary Table 1) shows the existence of all the elements of Y, Yb, and Er which indicates that the RE ions were present in our nanoparticles. It should be noted that due to the significant overlap of the L- and M-edges of Y, Yb, and Er, it was difficult to deconvolute the EDX spectrum peaks to reflect the concentrations of Y, Yb, and Er, especially considering the low Yb, Er dopant concentrations. Therefore, the measured Y compositions would also include contributions from the Yb and Er dopants. Figure 1c shows the steady state emission spectrum of our UCNs upon excitation at 975 nm. The emission peaks are attributed to the ^2^H_11/2_ → ^4^I_15/2_ (~525 nm), ^4^S_3/2_ → ^4^I_15/2_ (~540 nm), ^4^F_9/2_ → ^4^I_15/2_ (~654 nm) transitions of the rare earth dopant, Er^3+^. The undoped shell effectively eliminates any quenching that arises from surface defects or quenching groups (e.g., OH and CH_2_ groups), and shields the Er^3+^ emitting centers in the core from external contaminants and organic surfactants. Thus, the UC efficiency increased significantly upon coating the core with an undoped shell (see Supplementary Fig. 3). Figure 1d shows the time-resolved luminescence spectrum of our UCNs measured at 540 nm upon excitation at 975 nm. The luminescence decay curve was fitted using a single exponential equation of *I* = *I*_*0*_ exp(−*t*/*τ*), where *I*_*0*_ is the initial emission intensity at *t* = 0 and *τ* is the fitted decay lifetime. The estimated decay time of as-synthesized UCNs was estimated to be ~0.44 ms for the ^4^S_3/2_ → ^4^I_15/2_ (~540 nm) transition. The decay time characterizes the radiative and non-radiative relaxation of excited states. Generally a long decay time indicates low non-radiative losses and high emission efficiency. Thus, the long decay time of ~0.44 ms for our UCNs compared to the reported value of ~0.20 ms for a similar UCN with the same crystal phase suggests that we have prepared highly efficient UCNs using our synthesis method^50^.

### Preparation and characterization of nanocomposite film

**Figure 2:**
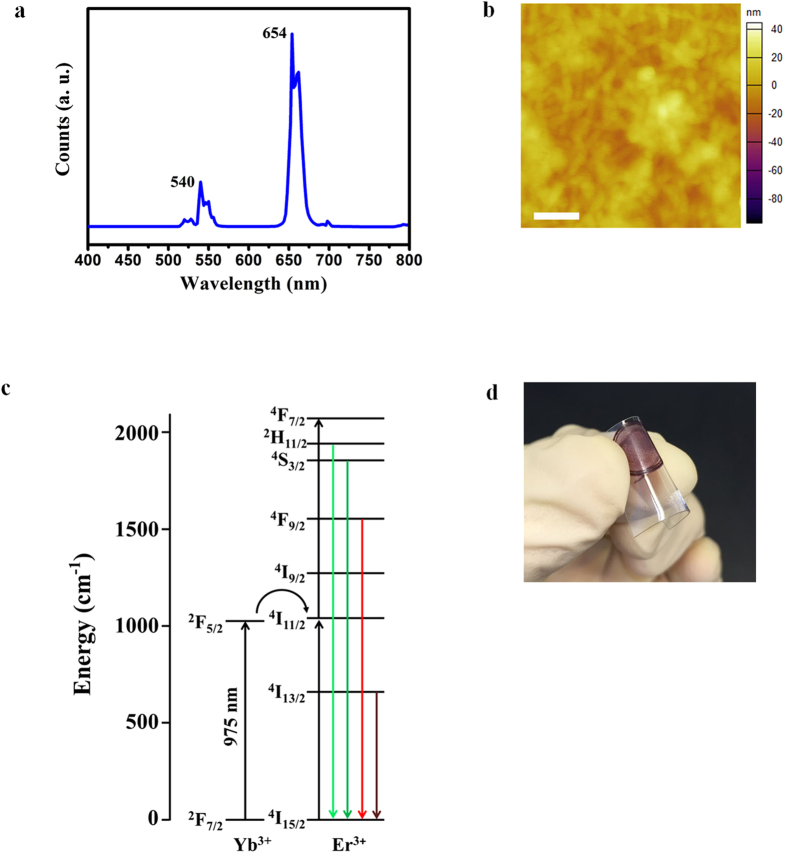
(**a**) Photoluminescence spectrum of UCN-P3HT nanocomposite film. (**b**) Atomic force microscopy (AFM) image of our UCN-P3HT nanocomposite film. Scale bar, 200 nm. (**c**) Schematic of electronic transitions in NaYF_4_:Yb,Er core-shell nanoparticles upon 975 nm excitation. (**d**) Photograph of nanocomposite film on a flexible polyethylene terephthalate (PET) substrate.

The nanocomposite film was fabricated by spin coating using a solution consisting of UCNs dispersed in a P3HT solution. The steady state emission spectrum of our nanocomposite film is shown in Fig. 2a. The intensity of green emission at 540 nm decreases relative to that of red emission at 654 nm. The integrated intensity ratio of green to red emission of our as-synthesized UCNs and nanocomposite film is shown in Supplementary Fig. 4. The green-to-red ratio decreases from 0.56 for NaYF_4_:Yb,Er core-shell nanoparticles to 0.23 for nanocomposite film. The observed decrease in green emission intensity is attributed to the preferred absorption of the green emission by P3HT. The surface of the obtained nanocomposite film was observed using both AFM (Fig. 2b) and SEM (Supplementary Fig. 5). The root mean square (RMS) surface roughness is estimated to be ~7.79 nm. The relatively small RMS value suggests that the surface is highly uniform. The uniform surface texture also indicates that the UCNs were homogenously dispersed within the P3HT film. The electronic transitions of our UCNs are shown in Fig. 2c. Upon NIR excitation, the Yb^3+^ ions absorb NIR photons through the ^2^F_7/2_ → ^2^F_5/2_ energy transition and subsequently undergo energy transfer to nearby Er^3+^ ions. Through energy transfer and cross-relaxation pathways, visible light is emitted through the ^4^S_3/2_ → ^4^I_15/2_ (~540 nm) and ^4^F_9/2_ → ^4^I_15/2_ (~654 nm) transitions of Er^3+^ ions. P3HT which has a bandgap of 1.9 eV results in corresponding absorption for wavelengths less than 650 nm. Thus, the visible emissions from our UCNs are mostly absorbed by the P3HT molecules to generate electron-hole pairs or excitons. In this composite, the long excited-state lifetime of UCNs would be most beneficial to the exciton generation process. Photocurrent is generated when a voltage bias is applied to the nanocomposite film upon exposure to NIR light. With more excitons generated, a larger photocurrent would be expected. Therefore, the performance of the photoconductor under IR light is partly dictated by the UC efficiency of our UCNs and the absorption efficiency of visible emission by the surrounding P3HT. To evaluate the possibility for making flexible device, P3HT film mixed with our UCNs was spin coated on a polyethylene terephthalate (PET) substrate as shown in Fig. 2d. By visual inspection, it is observed that our UCN-P3HT nanocomposite film adhered well with PET film and there is no visible breakage after multiple bending of the flexible substrate. The excellent adhesion demonstrates the outstanding potential of our UCN-P3HT film for flexible device fabrication. An image of flexible device is shown in Supplementary Fig. 6.

### Photoconductor structure and fabrication

**Figure 3:**
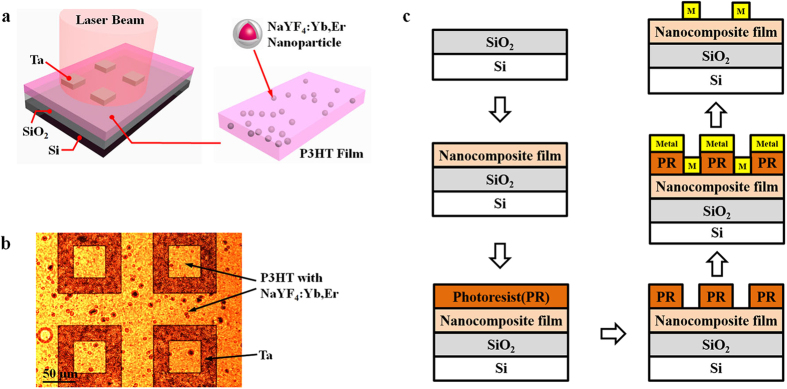
Photoconductor with the UCN-P3HT nanocomposite film. The thickness of each layer is not drawn to scale. (**a**) Schematic of our photoconductor device integrating our UCN-P3HT nanocomposite film. (**b**) Top-view microscope image of the device after all fabrication steps. (**c**) Schematic illustration of a detailed process flow of the photoconductor.

Figure 3a shows the schematic illustration of a photoconductor incorporating the UCN-P3HT composite film which was fabricated using the conventional semiconductor technologies in this work. Silicon wafer with silicon dioxide (SiO_2_) coated was used as the substrate material. After cleaning the top surface of SiO_2_ using isopropanol (IPA) and deionized (DI) water, the P3HT composite film with our UCNs was spin-coated onto the substrate followed by a 120 °C annealing for 3 minutes in air. The solution-based spin-coating process for our UCN-P3HT composite film is much more cost-effective when compared to the fabrication of conventional III-V materials which needs an expensive and time consuming epitaxial growth process. Next, lithography, metal deposition, and lift-off steps were done to form the metal pads on top of the composite film. Figure 3b shows the top-view image of the final device under an optical microscope. Figure 3c shows a detailed process flow of the photoconductor fabricated in this work. The whole process developed in this work shows a good compatibility to fabricate photoconductors with our UCN-P3HT composite film using traditional semiconductor processes, indicating a possibility of seamlessly integrating our UCN-P3HT composite film with the present semiconductor production line. Under excitation by near-infrared light, the NaYF_4_:Yb,Er nanoparticles emit photons which are absorbed by P3HT polymer film, resulting in a photocurrent generated between two metal pads under an applied voltage bias. The intensity of the photocurrent depends on the photon-to-electrical conversion efficiency of the UCN-P3HT nanocomposite film.

### Electrical performance of the photoconductor

**Figure 4:**
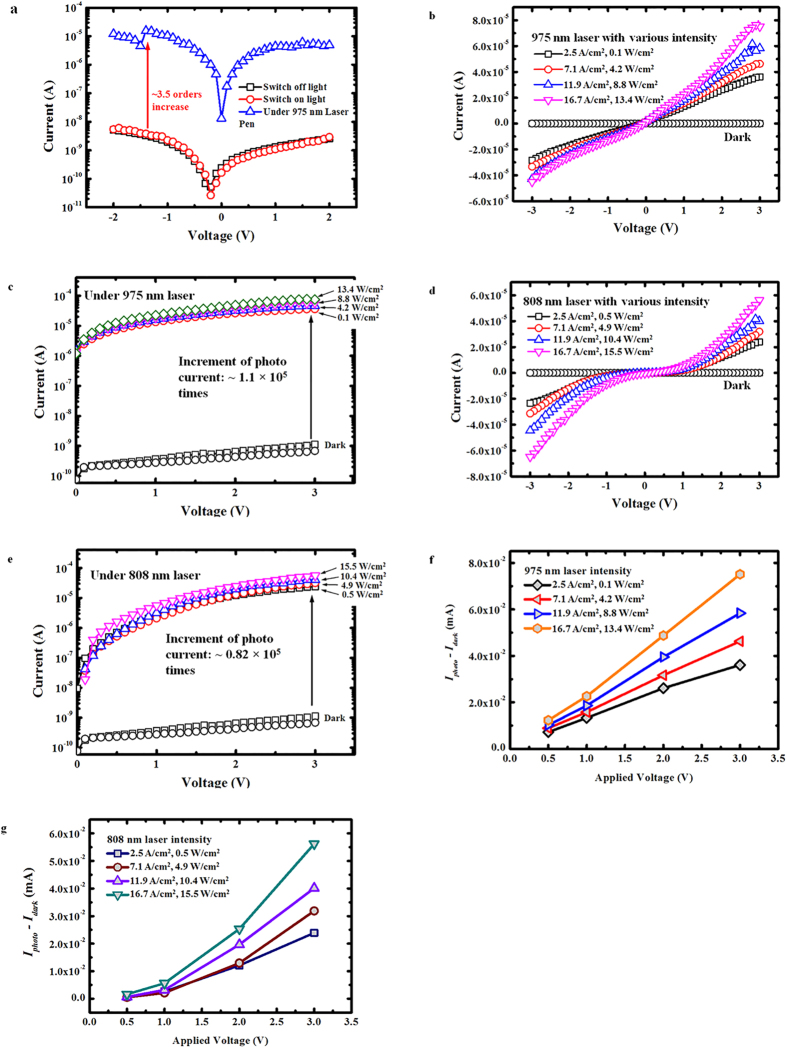
Electrical characteristics of photoconductors formed using the UCN-P3HT nanocomposite film. (**a**) *I–V* curve of photoconductor under illumination of a 975 nm laser pen. (**b**,**c**) Linear and log scale *I–V* curves of photocurrent under excitation of 975 nm laser with various power intensities. It shows a 1.1 × 10^5^ increment of photocurrent. (**d**,**e**) Linear and log scale *I–V* curves of photocurrent under excitation of 808 nm laser with various power intensities. It shows a 0.8 × 10^5^ increment of photocurrent. (**f**) Potential dependence of the increment of the photocurrent for 975 nm laser. (**g**) Potential dependence of the increment of the photocurrent for 808 nm laser.

For the photoconductor fabricated using the composite film, we measured the electrical characteristics of the devices under the illumination of lasers at different wavelength. Figure 4a shows the current-voltage (*I-V*) characteristics of the photoconductor under the illumination of a 975 nm wavelength laser pen. It is clearly observed that illumination at 975 nm leads to a considerable photocurrent increase of ~3.5 orders when compared to dark current. The significant enhancement can be attributed to the enhanced absorption and conversion of our UCN-P3HT nanocomposite film, where the efficiency of the UC emission processes of NaYF_4_:Yb,Er nanophosphors was a critical determinant. The sudden change of photocurrent at −1.5 V is caused by shaking of laser pen. The encouraging results that are obtained for the first-time for solution-processed photoconductions inspired further studies using excitation sources at other wavelengths (i.e. 975 nm and 808 nm) and power intensities using our photoconductors. In Fig. 4b and c, it was found that the photocurrent increased with the increase of the 975 nm laser power. A significant ~1.10 × 10^5^ times increment of photocurrent was achieved at the maximum illumination power (i.e. 13.4 W/cm^2^) using the 975 nm laser source. Compared to the results reported in the literature, this is a ~2.75 × 10^4^ times enhancement in terms of the increment of the photocurrent under the illumination of laser^47^. To examine the response of the photocurrent to incident laser power, the responsivity *R*_i_ of the photoconductor is calculated as *R*_i_ = *I*_photo_/*P*_in_, where *I*_photo_ is the photocurrent and *P*_in_ is the incident laser power. A responsivity of 7.6 A/W can be obtained from the photoconductor under an applied bias voltage of 3 V with 975 nm laser illumination of 0.1 W/cm^2^. This significant increase of photocurrent should be ascribed to the high upconversion efficiency of the as-synthesized UCNs whose decay time (~0.44 ms) is more than two times higher than the value (~0.20 ms) reported in the literature^47^ and the high loading (10 vol%) of UCNs in P3HT film. Both the high upconversion efficiency and high loading of UCNs provides much brighter visible emissions under the illumination of a 975 nm wavelength laser which can be subsequently absorbed by P3HT film to generate more electron-hole pairs or excitons. Thus large photocurrent increment can be observed when a voltage bias is applied. The large photocurrent increment further ascertains the compelling potential of using the nanocomposite film demonstrated in this work to advance the design of flexible optoelectronic devices. For measurements made using the 808 nm laser as shown in Fig. 4d and e, an obvious ~0.82 × 10^5^ times increment of photocurrent was found as well. A responsivity of 0.96 A/W can be obtained from the photoconductor under an applied bias voltage of 3 V with 808 nm laser illumination of 0.5 W/cm^2^. The increment would be associated to the optical characteristics of our UCNs, where NaYF_4_:Yb,Er nanophosphors exhibit a response upon excitation at both 975 and 808 nm. External quantum efficiency (EQE) which is defined as the number of electrons detected per incident photon can be expressed as *hcR*_i_/(*e*λ), where *h* is the Plank’s constant, *c* is the velocity of light, *e* is the electronic charge, λ is the excitation wavelength. EQE for the photoconductor at a bias of 3 V has been calculated as 966% and 122% for 975 nm laser illumination of 0.1 W/cm^2^ and 808 nm laser illumination of 0.5 W/cm^2^, respectively. It should be noted that our 975 nm laser source has a lower output power than that of the 808 nm laser source although the supplied current to the laser drivers is maintained at the same value, e.g. 3.5 A during the experiments. However, a higher photocurrent was measured upon illumination using the 975 nm laser than that of the 808 nm laser (at the same current). The higher photocurrent measured at 975 nm suggests that our nanocomposite film was more responsive and sensitive to the 975 nm laser compared to that of the 808 nm laser. Next, the effect of potential difference on the increment of photocurrent (i.e. *I*_*photo*_ − *I*_*dark*_) was investigated for both 975 nm and 808 nm lasers as shown in Fig. 4f and g. It was found that the increment of photocurrent became noticeably larger as the applied voltage on photoconductor increased. In addition, the saturation of photocurrent was not reached when the power intensity was at a maximum for both laser sources at 975 and 808 nm. Since photocurrent saturation has not been reached, the full potential of our UCN-P3HT nanocomposite film as a photoconductor has not been realized and a further enhancement can be expected at higher laser powers. The electrical characteristics of flexible devices are shown in Supplementary Fig. S7.

In summary, we have successfully fabricated a highly sensitive photoconductor using our UCN-P3HT nanocomposite film that was prepared using cost-effective solution-based processing method. In our nanocomposite film, the energy of near-infrared lights is converted to photoelectrons by UC process. For the first time, a ~5 orders increment of photocurrent was measured in this work upon near-infrared excitation. The photoconductor fabricated shows stronger photoresponse to 975 nm than that of the 808 nm laser source. Our approach and results demonstrated here would lead the designs and fabrications for next generation flexible and wearable near-infrared optoelectronic devices.

## Methods

### Materials

Regioregular Poly(3-hexylthiophene-2,5-diyl) (P3HT), was purchased from Rieke Metals Inc. (Nebraska, USA). Sodium trifluoroacetate (98%), yttrium (III) oxide (99.99%), ytterbium (III) oxide (99.9%), erbium (III) oxide (99.9%), trifluoroacetic acid (99%), toluene (99.8%), 1-octadecene (90%), oleic acid (90%) and oleylamine (70%) were purchased from Sigma-Aldrich (Sigma-Aldrich, St. Louis, MO). Chloroform (99.99%) was purchased from Aik Moh Chemicals Inc. All chemicals were used as received without any further purification

### Synthesis of NaYF_4_:Yb,Er core-shell nanoscrystals

The NaYF_4_:Yb,Er core-shell nanoparticles were synthesized by using a solvothermal decomposition method. The lanthanide trifluoroacetate precursors were prepared by dissolving stoichiometric ratios of lanthanide oxide powders in trifluoroacetic acid at 80 °C. In a typical experiment, a mixture of 0.78 mmol (CF_3_COO)_3_Y, 0.20 mmol (CF_3_COO)_3_Yb, 0.02 mmol (CF_3_COO)_3_Er and 1.5 mmol CF_3_COONa was dissolved in an organic solution containing 3.2 mL 1-octadecene, 2.5 mL oleic acid and 2 mL oleylamine in a 50 mL three-necks flask at 120 ^o^C under Argon gas flow. The obtained solution was heated to 330 °C and kept at this temperature for 1 h in the argon environment under vigorous stirring. Next, a shell solution containing 1 mmol (CF_3_COO)_3_Y, 1.5 mmol CF_3_COONa, 3 mL oleic acid and 2 mL oleylamine was added to enable the formation of core-shell particles. Upon completion of the reaction and after cooling, the synthesized nanoparticles were separated and washed three times in ethanol by centrifugation.

### Device fabrication

For the photoconductors, the silicon wafer with 1000 nm SiO_2_ was used as the substrate. The wafers were cleaned with isopropanol and deionized water for 2 mins and dried with nitrogen gas before use. P3HT solution (15 mg/mL) was prepared by dissolving P3HT in a mixed solvent of chloroform and toluene at a volume ratio of 1:1. Then NaYF_4_:Yb,Er core-shell nanoparticles were dispersed in the P3HT solution at a volume ratio of 10 vol% and the obtained nanocomposite solution was ultrasonicated before spin-coating. The nanocomposite solution was spin-coated onto the as-fabricated substrate with a spinning rate of 6000 rpm for 60 s, followed by annealing at 120 °C for ~3 min. After coating of the nanocomposite film on the substrate, the photoresist was coated using the spin coater at 3000 rpm followed by a 90 °C baking using hot plate for 1 min. Lithography was done using a Karl SUSS MA-600 mask aligner with a UV lamp. Post-exposure baking was done at 120 °C for 1 min using hot plate. Development was conducted to form the exposed area or desired pattern of the photoresist. The chemical residue was removed using deionized water and the sample was dried using nitrogen gas. A 100-nm-thick tantalum was deposited using an AJA physical sputtering system followed by lift-off process using acetone in an ultrasonic machine. Eventually, the tantalum metal pads were formed on the nanoparticle film and electrical characteristics could be measured on the above mentioned photoconductor structure. The dimension of the metal pads is 100 μm × 100 μm.

### Characterization

X-ray powder diffraction (XRD) pattern was measured on a D8 Eco Advance powder diffractometer (Bruker AXS Inc., Madison, WI) using Cu Kα radiation with wavelength of 1.5418 Å. Electronic micrographs were taken on a field emission scanning electron microscopy (FESEM, JSM-7600F, JEOL Ltd., JP). Particle morphology was measured using a JEOL 2010 transmission electron microscope operating at an acceleration voltage of 200 kV. Steady state luminescence spectra were measured upon excitation with a 975 nm continuous wave laser (CNI MDL-III-975, Changchun New Industries Optoelectronics Tech. Co. Ltd, China) using a FLS980 Fluorescence Spectrometer (Edinburgh Instruments Ltd., U.K.). To measure the time-resolved luminescence spectrum, the excitation source was modulated using an electronic pulse modulator to obtain excitation pulse at pulse duration of 30 μs with a repetition rate of 10 Hz. The laser powers of 975 nm continuous wave laser and 808 nm continuous wave laser (CNI MDL-H-808, Changchun New Industries Optoelectronics Tech. Co. Ltd, China) were measured using a laser energy meter (FieldMaxII-P, Coherent Inc.). The electrical characterization was performed using CascadeMicrotech Summit 11000 probe station and Keithley 4200-SCS Semiconductor characterization system.

## Additional Information

**How to cite this article**: Tong, Y. *et al.* Cost-Effective and Highly Photoresponsive Nanophosphor-P3HT Photoconductive Nanocomposite for Near-Infrared Detection. *Sci. Rep.* **5**, 16761; doi: 10.1038/srep16761 (2015).

## Supplementary Material

Supplementary Information
