# Supplementary material for: Cost-Effective and Highly Photoresponsive Nanophosphor-P3HT Photoconductive Nanocomposite for Near-Infrared Detection
Source: Sci Rep. 2015 Nov 16;5:16761. doi: 10.1038/srep16761 (PMC4645221; doi:10.1038/srep16761)
Supplement: Supplementary Information [file srep16761-s1.doc]

**Supplementary Information**

Cost-Effective and Highly Photoresponsive Nanophosphor-P3HT Photoconductive Nanocomposite for Near-Infrared Detection

Yi Tong†, Xinyu Zhao†, Mei Chee Tan*, and Rong Zhao*

†Authors contribute equally for this work.

*Corresponding Authors:

Address : Department of Engineering Product Development,

Singapore University of Technology and Design (SUTD), 8 Somapah Road, 487372, Singapore.

Email : [zhao_rong@sutd.edu.sg](mailto:zhao_rong@sutd.edu.sg) and meichee.tan@sutd.edu.sg

Web : <http://epd.sutd.edu.sg/faculty/zhao-rong/> and http://epd.sutd.edu.sg/faculty/tan-mei-chee/

Phone : +65 6499-4613 and +65 6499 4572

Fax : +65 6499-4613 and +65 6499 4572


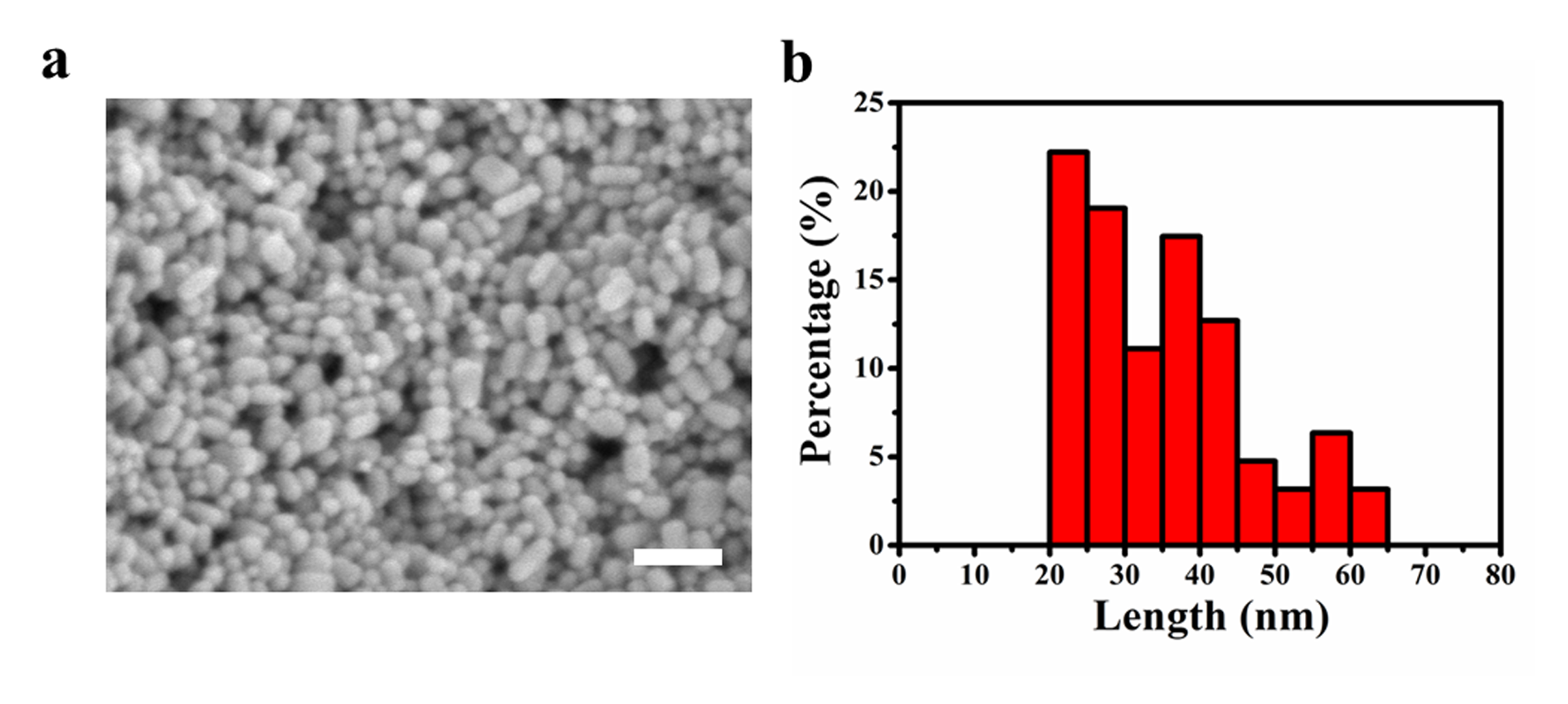


Figure S1. (a) SEM micrograph of NaYF_4_:Yb,Er core-shell nanoparticles, Scale bar, 100 nm. (b) Size distribution of NaYF_4_:Yb,Er core-shell nanoparticles.


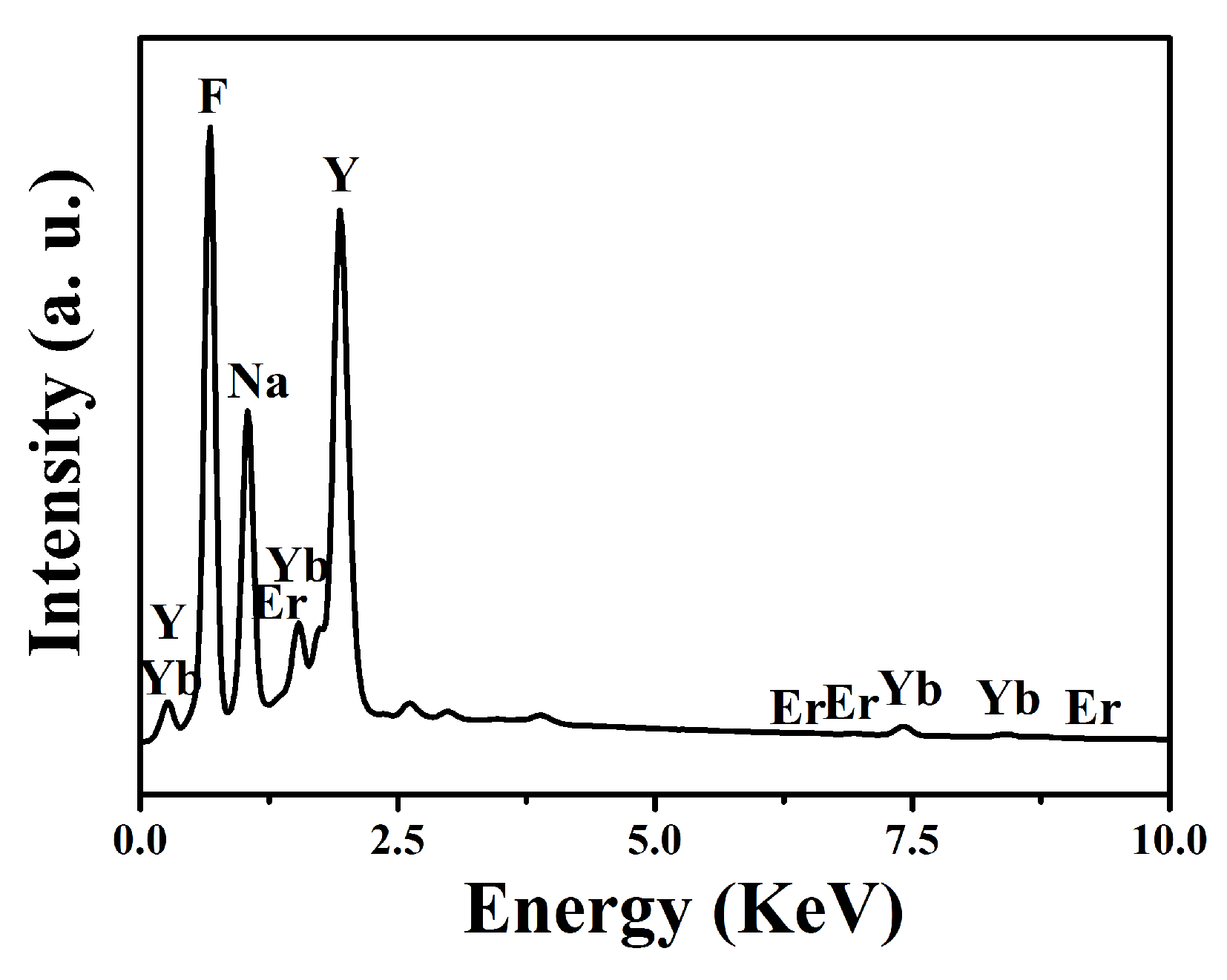


Figure S2. EDX spectrum of NaYF_4_:Yb,Er core-shell nanoparticles.

Table S1. Atomic percentage of each element of NaYF_4_:Yb,Er core-shell nanoparticles calculated from EDX spectrum.

| Element | Atomic % |
| --- | --- |
| F | 65.88 |
| Na | 18.95 |
| Y | 13.51 |
| Yb | 1.52 |
| Er | 0.14 |
| Total | 100.00 |


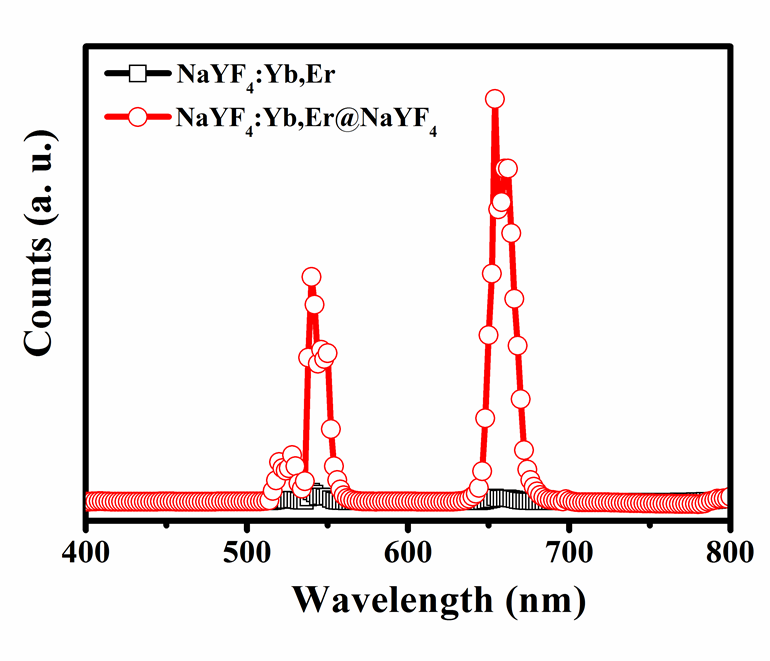


Figure S3. Photoluminescence spectra of NaYF_4_:Yb,Er core and core-shell nanoparticles. The integrated intensity in green and red emission was increased by 27 and 100 times respectively after covering a NaYF_4_ shell.


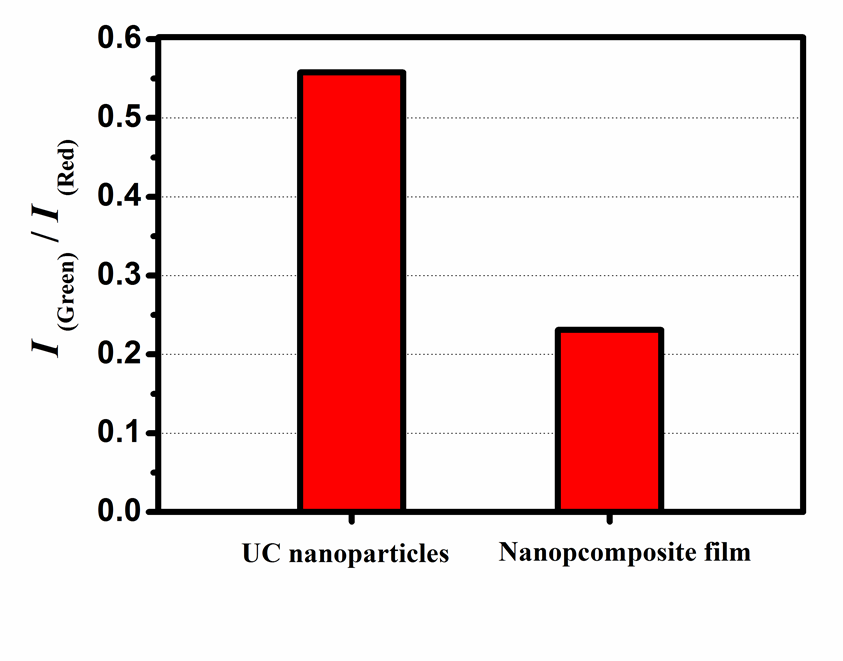


Figure S4. Integrated intensity ratio of green to red emission of NaYF_4_:Yb,Er core-shell nanoparticles and nanocomposite film.


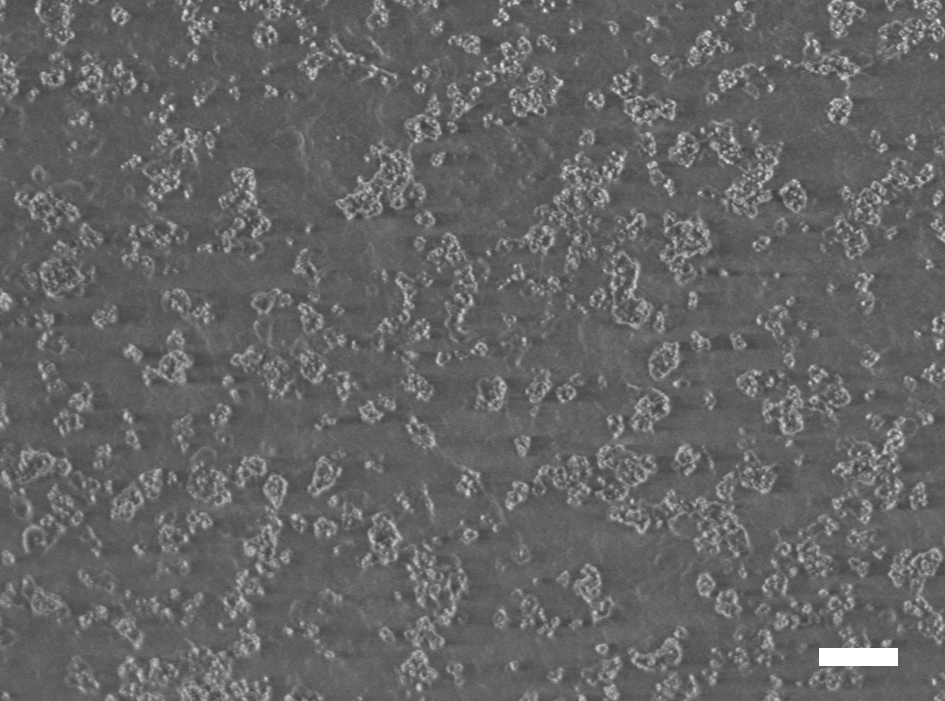


Figure S5. SEM image of nanocomposite film. Scale bar 1 μm.


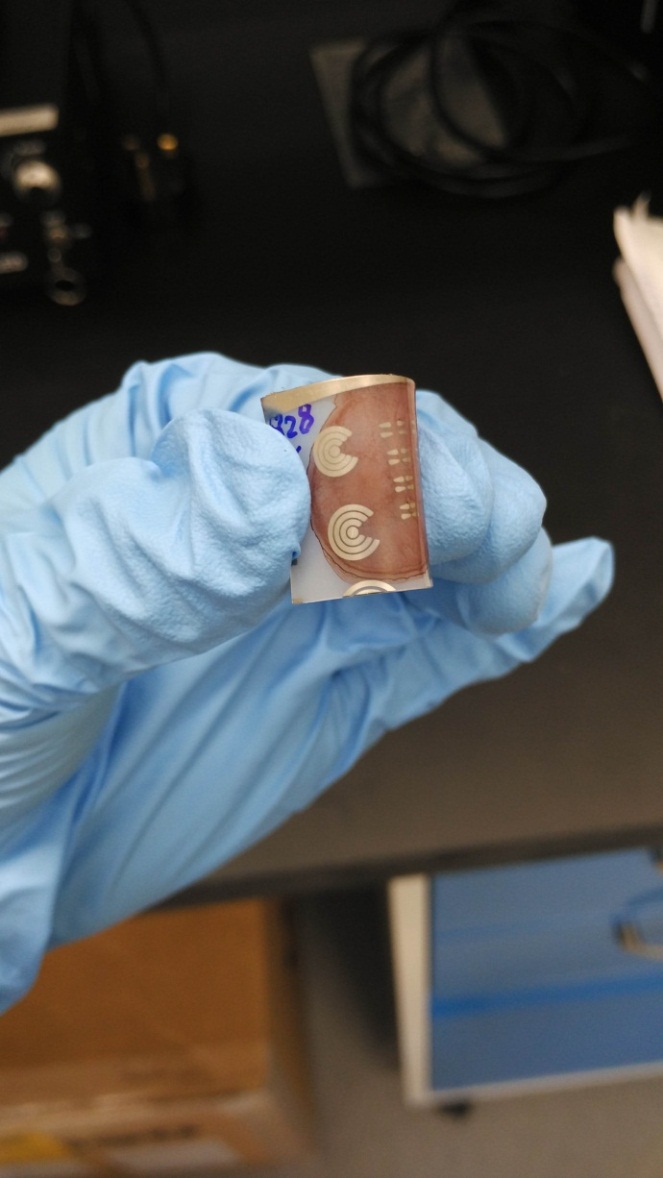


Figure S6. An image of flexible device using P3HT with NaYF_4_:Yb,Er on polyethylene.

Figure S7. Electrical characteristics of the flexible device under 975 nm laser illumination. The 975 nm laser intensities are 0 W/cm^2^, 0.1 W/cm^2^, 1.8 W/cm^2^, 4.1 W/cm^2^, 6.7 W/cm^2^, and 8.6 W/cm^2^, respectively. High photo current achieved with a responsivity of 0.62 A/W at 2 V.
